# A heterogeneous human immunodeficiency virus-like particle (VLP) formulation produced by a novel vector system

**DOI:** 10.1038/s41541-017-0040-6

**Published:** 2018-01-19

**Authors:** Joshua Pankrac, Katja Klein, Paul F. McKay, Deborah F. L. King, Katie Bain, Jason Knapp, Tsigereda Biru, Chanuka N. Wijewardhana, Rahul Pawa, David H. Canaday, Yong Gao, Sarah Fidler, Robin J. Shattock, Eric J. Arts, Jamie F. S. Mann

**Affiliations:** 1 Department of Microbiology and Immunology, University of Western Ontario, London, ON N6A 5C1 Canada; 2 Division of Infectious Diseases, Department of Medicine, Case Western Reserve University, Cleveland, OH 44106 USA; 3 Division of Medicine, Department of Infectious Diseases, Imperial College London, Norfolk Place, London, W2 1PG UK; 4 Department of Medicine, Imperial College London, London, UK

## Abstract

First identified as the etiological agent behind Acquired Immunodeficiency Syndrome (AIDS) in the early 1980s, HIV-1 has continued to spread into a global pandemic and major public health concern. Despite the success of antiretroviral therapy at reducing HIV-1 viremia and preventing the dramatic CD4^+^ T-cell collapse, infected individuals remain HIV positive for life. Unfortunately, it is increasingly clear that natural immunity is not, and may never be, protective against this pathogen. Therefore, efficacious vaccine interventions, which can either prevent infection or eradicate the latent viral reservoir and effect cure, are a major medical priority. Here we describe the development of a safe vaccine platform, currently being utilized in on-going prophylactic and therapeutic preclinical studies and consisting of highly heterogeneous virus-like particle formulations that represent the virus diversity within infected individuals. These VLPs contain no 5′LTR, no functional integrase, and have a severely mutated stem loop 1—thereby preventing any potential reverse transcription, integration, and RNA packaging. Furthermore, we demonstrate that these VLPs are morphologically identical to wild-type virus with polyvalent Env in a functional form. Finally, we show that the VLPs are antigenic and capable of generating strong immune recall responses.

## HIV: An investigatory vaccine system that mimics natural infection diversity

A system has been developed that allows the generation of HIV-mimicking particles which provoke an immune response from human cells. As HIV-1 remains pandemic, vaccination represents a current global research priority for prophylaxis, and could even offer a cure. Jamie Mann, of the University of Western Ontario, Canada, and an international research group have successfully created HIV-1 virus-like particles (VLPs) designed to prime immune systems against a diverse range of viral envelope protein conformations. The VLPs produced are morphologically near-identical to the virulent ‘wild-type’ HIV, and successfully stimulated the production of IFN-γ and Granzyme B from CD4^+^ and CD8^+^ T immune cells in vitro, in cell cultures taken from HIV-infected volunteers. The VLPs appeared safe in vitro; however, further investigation will confirm their safety profile in humans. The authors are currently investigating different forms of these VLPs as potential anti-HIV therapeutics.

## Introduction

Since its identification as the etiological agent behind Acquired Immunodeficiency Syndrome (AIDS), human immunodeficiency virus type 1 (HIV-1) has become a global pandemic and major public health concern. Despite the success of antiretroviral therapy (ART) at reducing viral loads and preventing CD4^+^ T-cell loss, infection endures owing to the establishment of a transcriptionally silent latent reservoir in CD4^+^ T cells.^1^ As natural immunity is not protective against HIV-1, the development of an effective vaccine strategy remains the best countermeasure to the advancing pandemic.

Despite a lack of natural immunity against HIV-1 infection and few correlates of protection to guide vaccine development, it is anticipated anti-HIV-1 vaccines should elicit potent cellular and humoral immune responses. Apart from the modest protection observed in the RV144 trial, HIV vaccines have failed to induce any protective efficacy in clinical trials.^2,3^ Accordingly, there is a renewed focus on therapeutic HIV vaccine development and the elimination of cell-associated or cell-free HIV in patients treated with ART. Thus it is a priority to develop improved vaccine immunogens that can activate both cellular and humoral immune responses with a view to not only protect against HIV acquisition but also to eliminate viral reservoirs such that viral remission or even a cure is achieved. A significant impediment to vaccine efficacy is thought to be the sheer HIV-1 sequence diversity.^4^ A preventative vaccine must protect against HIV strains/subtypes circulating in multiple geographic regions, while therapeutic vaccines must elicit an immune response that overcomes the high intra-patient sequence diversity and any immune escape mutations. Polyvalent Env vaccines are one approach designed to overcome the viral diversity and involves the use of heterologous HIV-1 Env mixtures to expose developing immune responses to diverse Env conformations.^5–8^ Nonetheless, a monovalent, let alone polyvalent, native Env structure is difficult to achieve outside of the HIV particle. Polyvalent Env vaccines may provide vaccine breadth and harness the elusive neutralizing antibody response, which has both therapeutic and preventative benefits.^9–11^ This does not discount that non-neutralizing antibodies may play a vital role in vaccine-mediated protection.^12^ From another standpoint, eliciting broad cellular immune responses through vaccination is also highly desirable considering cytotoxic T lymphocyte (CTL) responses can reduce viral loads, thereby contributing to the elimination of productively infected cells.^13^

In pursuit of more efficacious HIV vaccines, efforts have focused on virus-like particles (VLPs), due to their capability of presenting native Env spikes on their surfaces, as well as their capacity to be endocytosed and presented to T cells in the context of both major histocompatibility complex class I and II.^14^ VLPs are defined as viral particles (VPs) that have undergone protein and lipid self-assembly to generate non-infectious VPs devoid of any viral genetic material. Furthermore, VLPs may be morphologically indistinguishable from wild-type infectious virus and present the entire HIV proteome as antigen. Currently, there are four licensed VLP-based prophylactic vaccines providing protective immunity against influenza, Hepatitis B, and Human Papilloma virus infection.^14^

Herein we describe a safe, chimeric HIV-1 VLP vector system, capable of accommodating near full-length HIV genomes and captures the HIV diversity present within patient samples. The vector system was designed to generate VLPs for testing as anti-HIV therapeutic and prophylactic vaccine purposes.^15^ Viral RNA from plasma of HIV-infected volunteers was reverse transcribed and cloned into a DNA vector for yeast-based recombination/gap repair. Full-length patient-derived HIV-1 genomes were mutated during the cloning process to stop reverse transcription, integrase (IN) activity, and genomic RNA packaging into VLPs. The VLPs express the processed HIV-1 proteome, were morphologically indistinguishable from HIV VPs, and were capable of stimulating both CD4 T cell and cytotoxic responses in heterologous patient samples. Finally, the VLPs were combined to form a highly diverse vaccine formulation called Heterologous Clade B Activating-Vector (Het_B_ACT-VEC), which shared the phenotypic and antigenic properties of the aforementioned VLPs.

## Results

### Yeast-based gap/repair recombination to clone full-length HIV genomes from patients

Here we describe the ease of full genome cloning to produce patient-derived VP (containing the viral genome) and VLPs (lacking the viral genome), the latter of which is currently being tested as a therapeutic vaccine (JFS Mann, manuscript submitted). In addition, we will describe the representative sampling of the HIV population from plasma of infected patients into DNA vectors used to produce the vaccine constructs. Ultimately, the VP of different patients can be combined to generate heterogenous, near full-length, multivalent vaccines for both therapeutic (as part of amfAR preclinical studies) and preventative modalities (as part of the European HIV Vaccine Initiative).

**Fig. 1:**
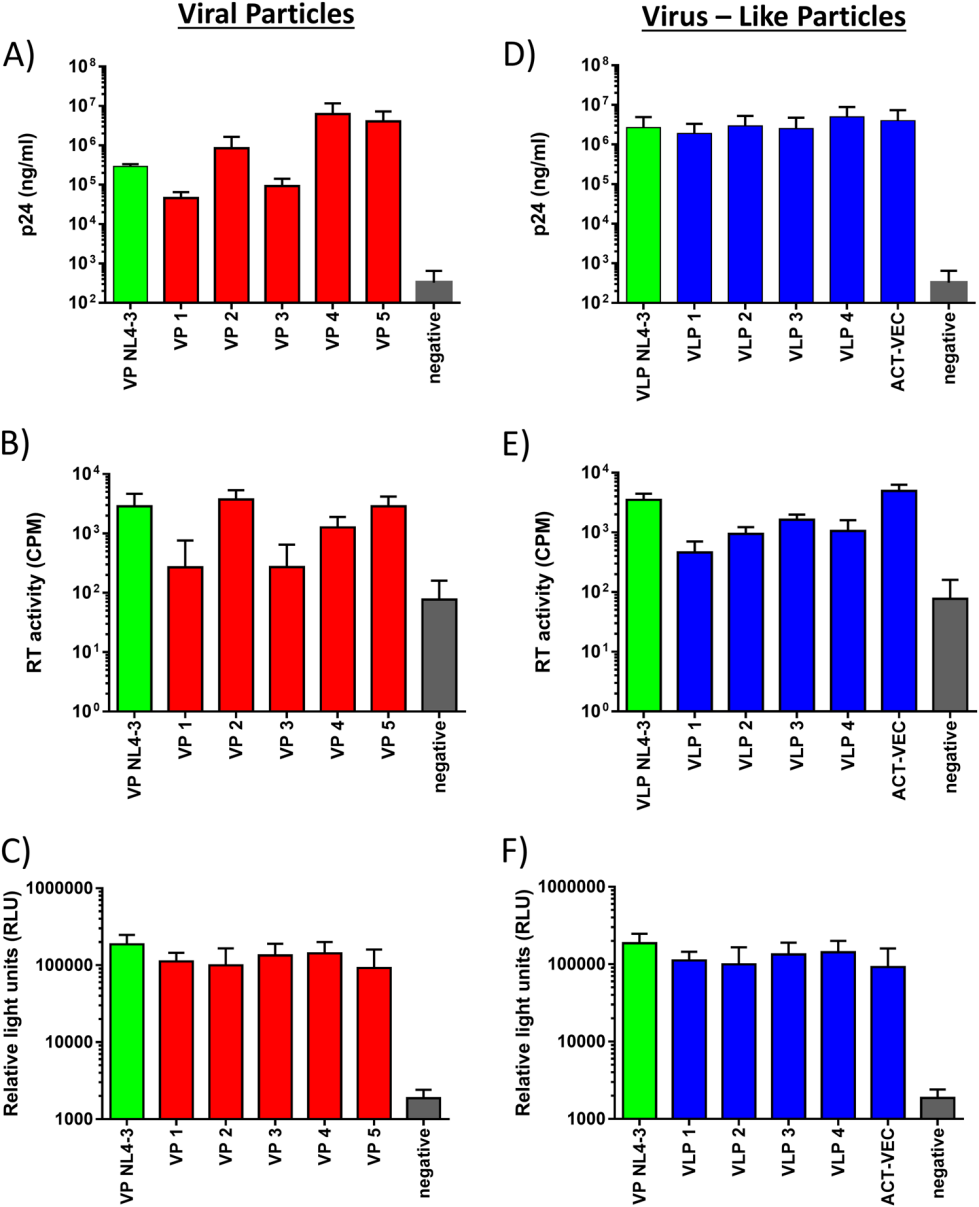
Viral particle (VP) and virus-like particle (VLP) formulations express similar vial protein concentrations. **a**, **d** pREC_nfl VP (red) and pREC_nfl_dS.1/mutIN VLP (blue) DNA constructs were used to transiently transfect 293T cells in 24-well tissue culture plates for 48 h. After 48 h, culture supernatants were assessed for viral p24 production using a p24 ELISA Kit. Results shown represent mean p24 values (+/− SEM). **b**,** e** Culture supernatants were also harvested to asses VP and VLP reverse transcriptase activity in counts per minute (CPM) using an in-house radioactive RT assay. **c**,** f** To demonstrate the presence and functionality of HIV Env on VP and VLP, an HIV-1 co-receptor tropism assay was used. The 293T cells were transfected with the VP and VLP pREC-nfl plasmids and mixed with CD4^+^/CCR5^+^ U87 cells harboring the pDM128FLUC plasmid. Cell fusion elicits luciferase expression if the 293T cells express functional HIV-1 gp120/gp41 Env glycoproteins, Rev, and Tat from the pREC-nfl vectors. Results are represented by mean relative light units (RLU) (+/−SEM) with background luminescence subtracted from positive and negative results

A total of five HIV^+^ plasmas from infected volunteers, diagnosed at chronic stage of infection, were used to generate our vaccines. Samples from chronic disease were chosen to clone VP preparations as they exhibit extensive diversity compared to acute samples or transmitted/founder clones and therefore may provide better vaccine breadth. Following reverse transcription (RT), we used external-nested PCR to amplify the full genome of HIV in two halves, overlapping by 113 bp within IN. These overlapping 5′ and 3′ patient-specific HIV-1 DNA fragments were transfected into *Saccharomyces cerevisiae* along with linearized pREC-nflΔgenome/URA3. Following a double recombination event in yeast, all resulting colonies were harvested to acquire maximum viral diversity—a process well described in multiple articles^16–18^ (Fig. S1). The DNAs were then transfected into 293T cells to produce VPs. As shown in Fig. 1a–c, we detected significant levels of HIV-1 capsid p24 and RT activity in the cell-free supernatants with each of the pREC-nfl plasmids containing the genomes of the patient-derived HIV (VP1, VP2, VP3, VP4, and VP5).

We also assessed and verified the presence of p24 and RT activity in 293T cells transfected with pREC-nfl plasmids (Fig. 1a, b). Env expression was confirmed using a viral tropism (Veritrop) assay (Fig. 1c). The Veritrop assay was done by transfecting 293T cells with the pREC-nfl plasmids and mixing the cells with CD4^+^/CCR5^+^ U87 cells, which harbor the pDM128FLUC plasmid as previously described.^19^ Here cell fusion and light emission occurs when 293T cells express functional HIV-1 gp120/gp41 Env, Rev, and Tat from the pREC-nfl vectors. Env binds CD4^+^/CCR5^+^ and mediates 293T/U87 cell fusion, permitting luciferase protein expression. All VP and VLP pREC-nfl vectors had similar levels of light emission, indicative of similar levels of Env, Rev, and Tat.

### Genetic diversity of patient-derived HIV-1 genomes with the pREC nfl vector

**Fig. 2:**
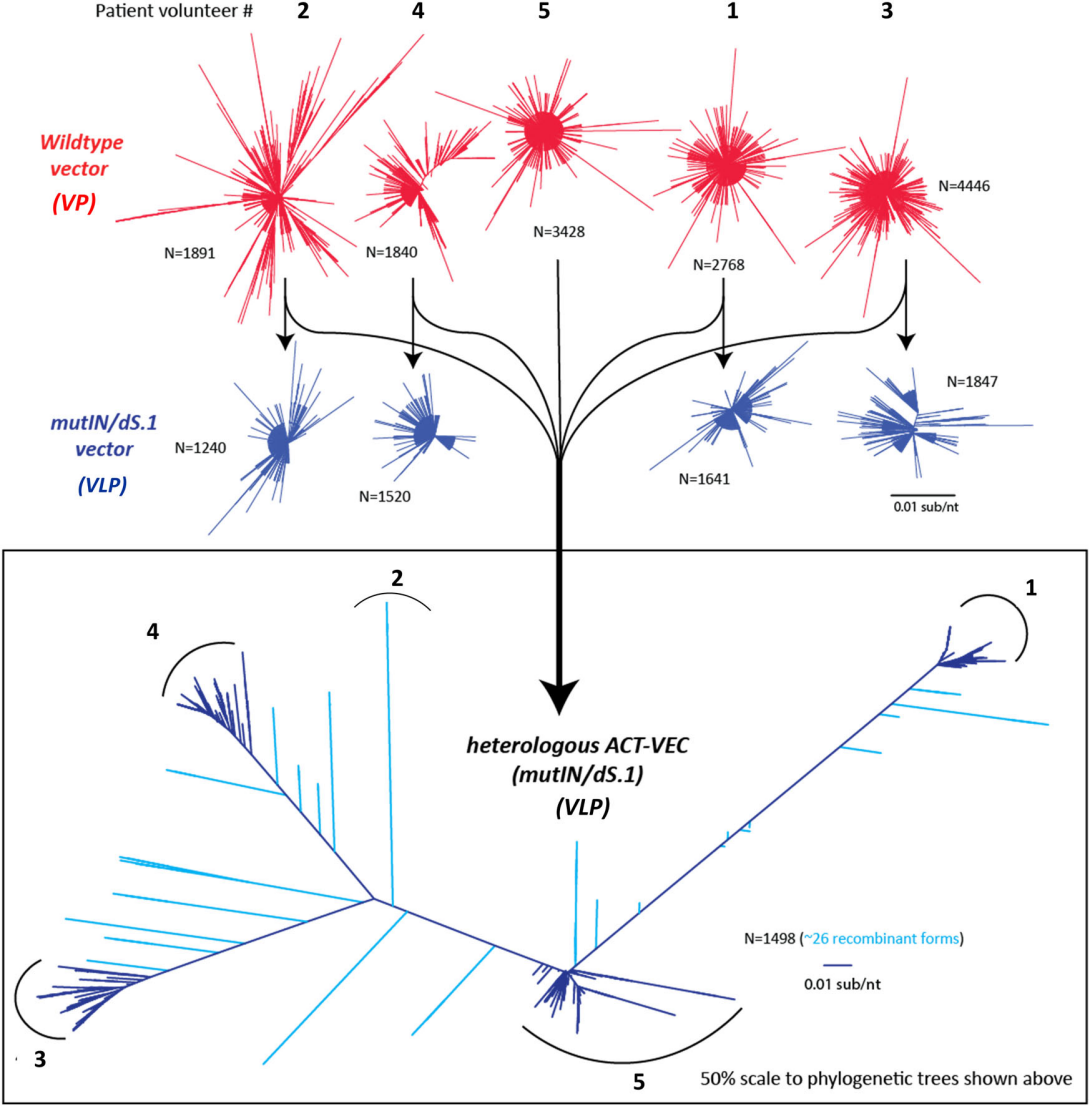
VPs and VLPs are genetically diverse preparations. Neighbor joining trees of nucleotide sequences were generated with MEGA6 and visualized with FigTree 1.4.2 to highlight sequence heterogeneity. Phylogenetic trees were reconstructed for viral particles (red) and for virus like particles (blue). VLP pREC_nfl DNAs were combined to generate Het_B_ACT-VEC

The sheer genetic quasi-species of HIV-1 present within infected individuals, owing to a combination of low-fidelity RT activity and genetic recombination, is a significant obstacle in the pursuit of viable prophylactic and therapeutic HIV vaccines. Thus a successful vaccine might need to be sufficiently “rich in diversity” to have adequate protective coverage. Several vaccine studies have evaluated heterologous, polyvalent, and sequential vaccine strategies to promote B-cell affinity maturation and enhanced antibody production. Yeast-based recombination/gap repair is a highly efficient cloning technique and can yield hundreds of yeast colonies carrying pREC clones with HIV genomes. To determine the relative genetic bottlenecks in the cloning strategy, we RT-PCR or PCR amplified the C2-V3 env fragment from the patient plasma, from the recombined and purified pREC-nfl vector, and from the gRNA contained in the VLPs. These env PCR products were then subjected to next-generation sequencing (NGS) and analyzed using the methods described previously.^20^ Similar topologies in maximum likelihood phylogenetic trees, similar genetic distances, and similar numbers of unique clones (>1% of the total sequence reads) for the plasma, plasmid, and WT Env population were seen from each patient sample (Fig. 2 and Table S2).

### Reducing genomic RNA packaging and inactivating IN

**Fig. 3:**
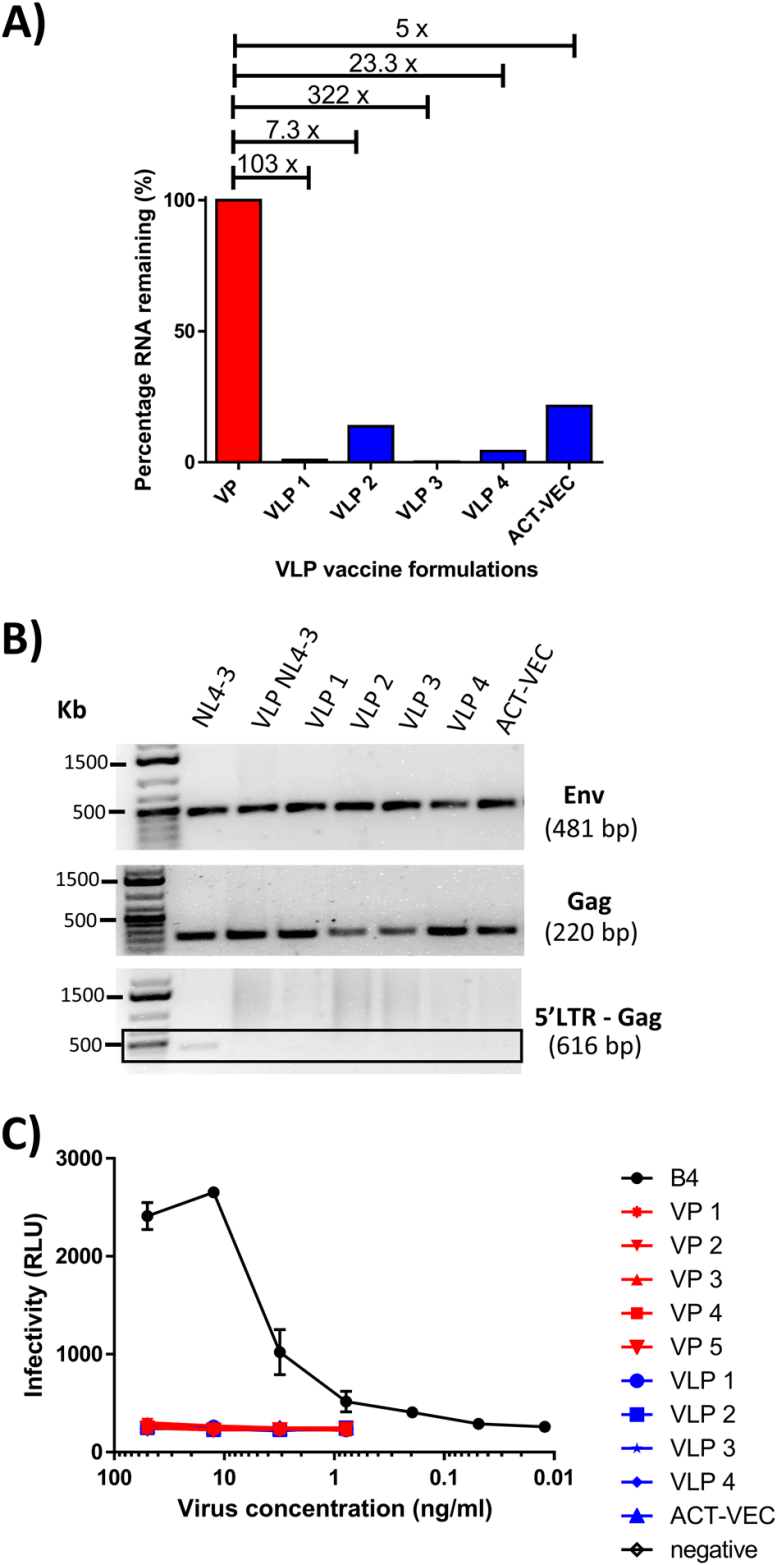
Virus-like particle formulations are non-infectious due to engineered RNA packaging defects and deletion of the HIV-1 5′LTR. **a** RNA packaging knockdown in individual VLP formulations (VLP1-4 + Het_B_ACT-VEC) were compared against near-full length viral particles (VP) formulations lacking mutations in the RNA packaging sequence by first isolating viral RNA and then by qRT-PCR using a gag primer set. **b** The VLP encoding pREC_nfl DNAs were evaluated for the presence/absence of HIV-1 gag, env, and 5′LTR by PCR and gel electrophoresis using gag, env, and 5′LTR—gag primer sets. Samples derived from the same experiment and gels were processed in parallel. **c** VP (-dS.1/mutIN) and VLP (+dS.1/mutIN) formulations were compared to infectious B4 virus for infectivity using luciferase TZM-bl cells. Infectivity results are represented by relative light units (RLUs). Luciferase quantification was done in a Synergy H4 Hybrid microplate reader using 50 μl of luciferase assay reagent

Full genome amplifications of the five patients were repeated but using primer sets to disrupt the gRNA packaging signal and/or the IN active site (Fig. S1). The 5′ primer of fragment 1 contained either 9 point mutations in stem loop 1 (698C>T, 718C>G, 719G>T, 720G>C, 721C>G, 722AT, 723A>T, 724G>C, and 731G>A, herein designated dS.1) or a 33 bp deletion within stem loop 3 (nucleotides 755–787, herein designated ΔSL3) as described previously by Clever and Parslow.^21^ This work was initially carried out to identify the minimal gRNA packaging element of HIV-1 using HXB2 pseudotyped with amphotropic murine leukemia virus. However, in our system it was necessary to confirm that these mutations would reduce gRNA encapsidation in our patient-derived VLP. Thus our VLPs would be inherently safer vaccine constructs compared to our VP formulations. Initially, the dS.1 mutation and ΔSL3 deletion were introduced into the pREC-nfl_NL4-3_ and used to transfect 293T cells (Fig. S2a, c, e). Resulting particles were harvested and purified from cell-free supernatants and found to have 142- and 11.6-fold less gRNA with the dS.1 and ΔSL3 mutations, compared to NL4-3-VPs. This work was further verified using HIV-1 clade C Env 1086 (Fig. S2b, d, f). We then mutated the patient-derived viruses, VP3, VP4, and VP5 and again found that the dS.1 mutations impaired gRNA packaging more so than the ΔSL3 mutations (Fig. 3a and Fig. S3c).

With evidence that dS.1 reduced gRNA packaging in primary vaccine particles, we proceeded to introduce both the dS.1 mutation and the RRK>AAH IN mutations via primer-related replacement during PCR amplification of the 5′/upstream genome half and the IN mutations into the 3′/downstream half of the genome. The two overlapping PCR products representing the halves of the genome with these mutations were then cloned into our pREC vector by yeast recombination/gap repair as described above. Following plasmid purification and particle production from transfected 293T cells, we tested these dS.1/mutIN-mutated VLPs for CAp24/Gag content by enzyme-linked immunosorbent assay (ELISA) and western blot, encapsidated gRNA by real-time RT-PCR, endogenous RT activity using exogenous poly(rA)oligo(dT) template, or the endogenous gRNA template (Fig. 1), and finally, IN activity by using VLPs soaked in lysis buffer containing radiolabeled substrates for dinucleotide cleavage by IN (data not shown). The addition of the RRK>AAH IN mutations, removal of the 5′ long terminal repeat (LTR), and introduction of the dS.1 mutations did not impact Gag/CAp24 levels or RT activity on an exogenous template in the VLP preparations. However, none of these mutated VLPs had dinucleotide cleavage activity/end processing as mediated by functional IN, as observed and compared with wild-type HIV particles (data not shown).

Finally, we repeated the cell-to-cell fusion assay by transfecting 293T cells with the pREC-nfl vectors harboring the mutations with the patient-derived sequencing. Evidence of wild-type cell fusion suggest that Env is expressed from pREC and can bind to CD4^+^/CCR5^+^ on the U87 cells. Western blots show the presence of Env in cell-free supernatant (data not shown). Previous studies using a Vpr-blam construct expressed in trans and encapsidated into VLPs^22^ clearly show VLP entry into CD4^+^/CCD5^+^ cells and that the levels of VLP entry correlated with the levels of cell-to-cell fusion mediated by pREC-nfl (used to produce the same VLP).

### Vaccine VP and VLP are phenotypically identical to wild-type virus

**Fig. 4:**
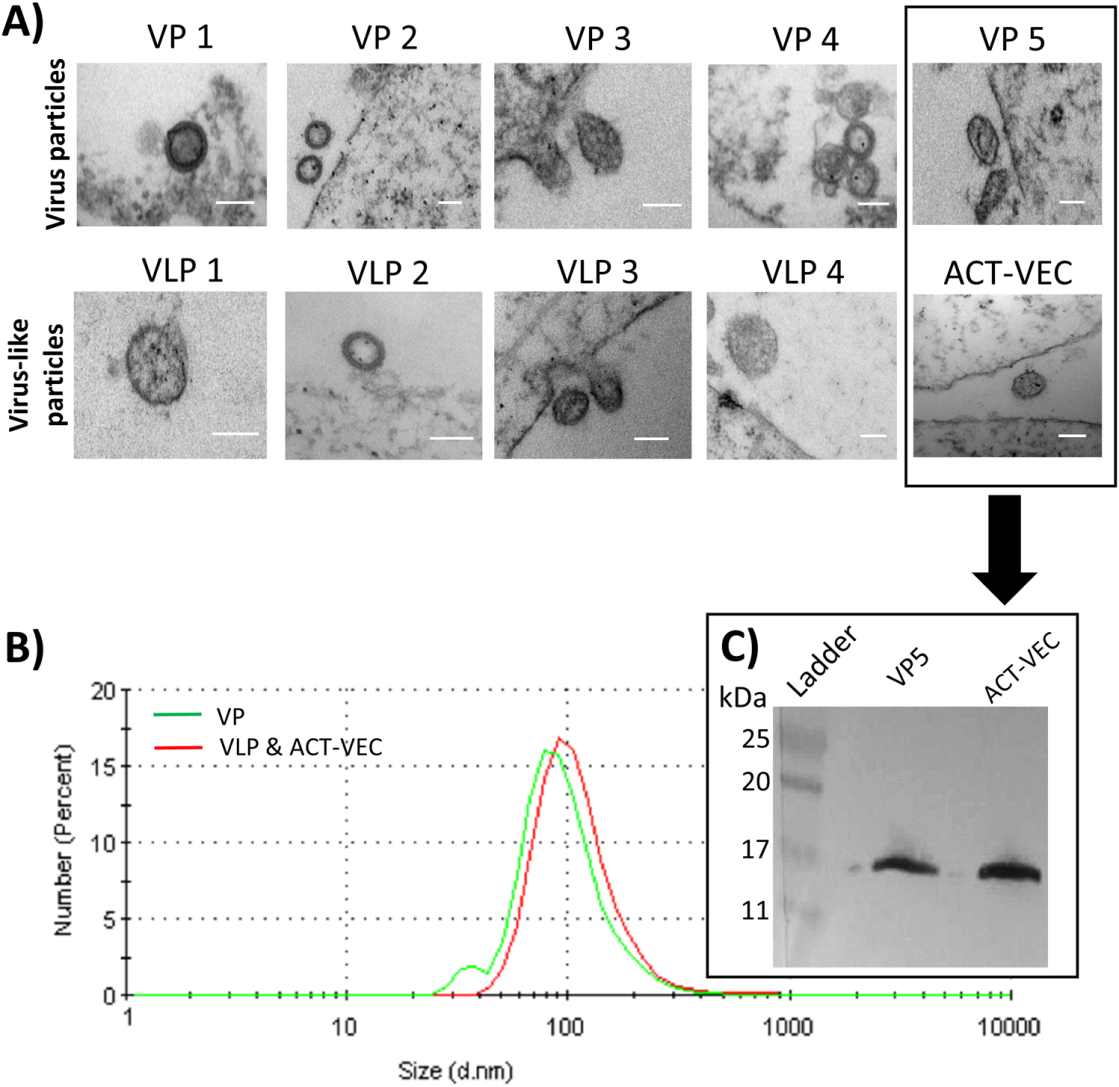
pREC-nfl derived VP and VLPs share are morphologically similar to wild-type virus. **a** 293T cells were transfected with pREC_nfl plasmids encoding VPs (−dS.1/mutIN) and VLPs (+dS.1/mutIN). Samples were fixed and embedded in Resin-Araldite Embed 812 before imaging via transmission electron microscopy (Philips CM10 TEM). White scale bar is 100 nm. **b** Purified VP and VLP preparations were analyzed by dynamic light scattering at 25 °C using a Malvern Zetasizer Nano (Malvern Instruments Ltd). The intensity of the laser light scattered by the sample preparations was detected at 90° to the incidence beam. Data were analyzed using the Malvern software. **c** Purified VPs and VLPs were assessed for evidence of protease cleavage of the gag-pol polyprotein by anti-p17 western blot

As described above, the dS.1/mutIN VLPs and wild-type VPs contained similar amounts of Gag and Env proteins and similar levels of RT activity. However, the dS.1/mutIN VLPs lacked IN activity, HIV-1 gRNA, and the ability to initiate and reverse transcribe (–) strand strong stop DNA. However, the general morphology of the VLPs compared to wild-type HIV is unknown. Thus we performed transmission electron microscopy (TEM) on 293T cells transfected with our pREC_nfl constructs. All VPs and dS.1/mutIN VLP pREC-nfl DNAs produced vesicular structures around the cells with some appearing to bud from the cell surface (Fig. 4a). These circular structures contained electron-dense membrane layers and were ~100 nm in diameter, akin to HIV particles. Tetherin is constitutively expressed in restrictive human cells and cell lines such as HeLa, H9, Jurkat, Molt4, primary T cells, and primary macrophages.^23^ As Tetherin activity is absent in the 293T producer cell line, we did not expect nor did we observe any VPs or VLPs “tethering” on the surface of transfected cells. However, since the full HIV-1 proteome is produced by transfection with pREC_nfl DNAs, we suspect that HIV-1 Vpu is produced and should downregulate/degrade of BST2/Tetherin^24^—supporting the use of the pREC-nfl as DNA vaccine vectors. As the VPs and VLPs were imaged while budding or shortly after budding from cell membranes, these particles are immature and therefore the absence of noticeable capsid within the particles was expected. As p24 and protease cleaved p17 (Figs. 1 and 4c) are readily detected in all preparations, we would expect an electron-dense capsid to form in mature VPs and VLPs.

The size of the purified VP and VLP was verified by dynamic light scattering (DLS). DLS depends upon Brownian motion and any resulting photon interference/deflection in liquid systems can be used to determine particle size and polydispersity. Following purification, the VP and VLPs had an average diameter centered around 100 nm (Fig. 4b), which agreed with the prior TEM measurements.

### Genetic diversity in the VPs and VLPs

Our previously formulated VP pREC-nfl derived from patient samples were compared to PCR amplified dS.1/mutIN pREC-nfl plasmid DNAs for their genetic diversity. The dS.1/mutIN VLPs do not harbor gRNA, so we cannot estimate genetic diversity within the patient-derived VLP preparations. We did compare the genetic diversity within the VP pREC and subcloned dS.1/mutIN pREC by PCR amplification and NGS of the C2-V3 of Env. The topology of the phylogenetic trees is similar for VP pREC-nfl and sub-cloned dS.1/mutIN pREC-nfl for each patient (Fig. 2). On average, each VP and VLP pREC_nfl contained 65 (range = 49–98) and 30.6 (range = 22–29) unique sequences, corresponding to an average of 0.006 (range = 0.00192–0.022) and 0.0178 (range = 0.0013–0.082) substitutions/nucleotide (Table S2).

### VLPs are non-infectious, devoid of 5′LTR, and have reduced viral RNA packaging

While inactivated (killed) whole VPs have been used to prevent a wide range of viral diseases, the use of AT-2 inactivated, ultraviolet (UV)-irradiated, whole HIV particles as a vaccine has been a concern due to safety but has recently undergone a phase I clinical trial evaluation showing no residual vaccine replication or any evidence of vaccine viral genetic material.^25^ Given the enhanced safety considerations in our preparation, it is important to note that previous generations of the VPs have been tested in mice, rabbit, and macaques with no adverse effects noted. Furthermore, the new generation of VLPs are currently being prepared for phase I clinical trials. Both the VP formulations and the dS.1/mutIN VLPs were unable to infect and replicate in a permissive luciferase expressing TZM-bl cell line. This contrasts with B4, an infectious subtype B chimeric virus (Env from a primary isolate placed into an NL4-3 backbone), which was readily able to infect this highly susceptible cell line in a concentration-dependent manner (Fig. 3c).

### Both VPs and Het_B_ACT-VEC can stimulate antigen-specific memory T-cell responses

The five volunteer-derived dS.1/mutIN pREC-nfl plasmid DNAs were all combined to generate a highly heterogeneous and polyvalent Het_B_ACT-VEC VLP preparation. This was used to transfect 293T cells and produce Het_B_ACT-VEC VLP formulations. We PCR amplified and sequenced the VP, dS.1/mutIN (VLP), and Het_B_ACT-VEC pREC-nfl sequences using NGS. Phylogenetic trees reveal a topology similar to a tree containing the population of all other pREC-nfl as expected (Fig. 2).

**Fig. 5:**
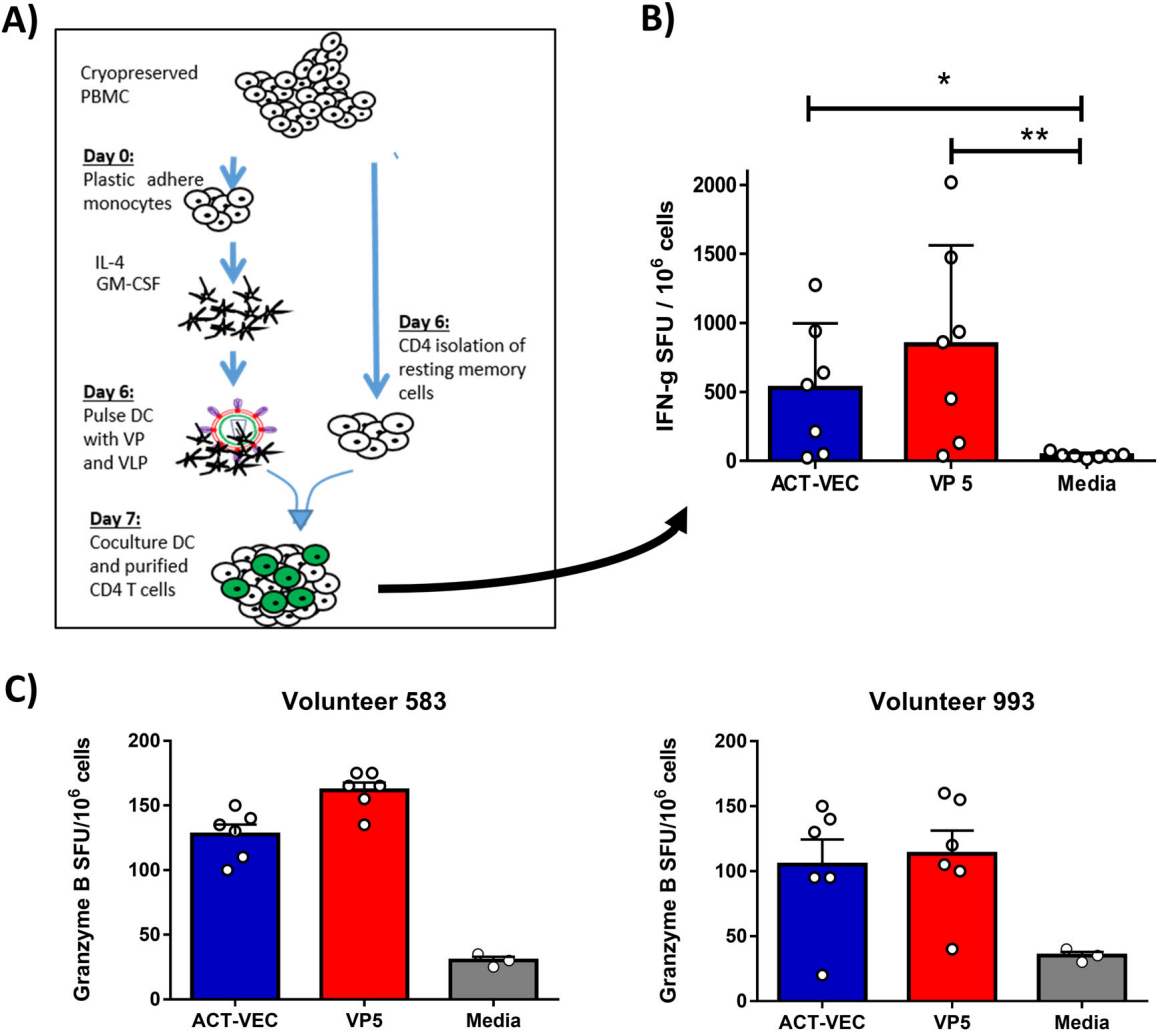
Purified VP and Het_B_ACT-VEC formulations are capable of human CD4^+^ T-cell activation in vitro. **a** PBMC from fully consented HIV^+^ volunteers (*n* = 7) were used to generate monocyte-derived dendritic cells (MDDCs), which were pulsed overnight with Het_B_ACT-VEC VLP or VP5 and co-incubated with autologous purified CD4^+^ T cells. **b** Cells were cultured overnight in a human IFN-γ ELISpot assay and the IFN-γ spot-forming units were enumerated per 10^6^ CD4^+^ T cell using the ImmunoSpot S5 UV Analyzer and ImmunoSpot 5.0.9 software. Results shown are mean SFU/10^6^ CD4^+^ T cells (+/− SEM). **c** HIV-infected PBMCs from two randomly selected donors are shown, representing the ability of ACT-VEC and VP5 to elicit Granzyme B (GzB) cytotoxic responses using overnight culture in a GzB ELISpot. In all cases, an assay cutoff of 50 SFU/10^6^ cells was used. Mann–Whitney non-parametric *U*-test was used to determine inter-sample statistical significance. We considered *p* > 0.05 to be statistically significant. Graphics depicted in this figure were generated by the authors

To determine whether VP and Het_B_ACT-VEC were antigenic and capable of stimulating antigen-specific T-cell recall responses, we assayed the formulations in a monocyte-derived dendritic cell (MDDC)–CD4^+^ T-cell co-culture assay using cells derived from HIV-infected volunteers (Fig. 5a). Peripheral blood mononuclear cells (PBMCs) from seven volunteers were purified by negative selection to generate isolated, untouched CD4^+^ T cells with purity >95% (Fig. S4a). Patient-derived MDDCs were grown by plastic adherence and in the presence of interleukin (IL)-4 and granulocyte macrophages colony-stimulating factor (GM-CSF) for 6 days. Resulting MDDCs were checked for phenotypic markers of differentiation, such as HLA-DR, CD83, and CD209 (Fig. S4b). MDDCs were pulsed overnight with VP5 or Het_B_ACT-VEC before washing and co-culturing with autologous CD4^+^ T cells in a human interferon (IFN)-γ ELISpot assay (Fig. 5a, b). As shown, the VP5 (**p > 0.005) and the Het_B_ACT-VEC (*p > 0.05) were antigenic and generated significant numbers of spot-forming units (SFU)/10^6^ CD4^+^ T cells when compared to the unstimulated MDDC-CD4^+^ T cell co-cultures (Fig. 5b). No statistical difference in the generation of SFU was observed between VP5 and the Het_B_ACT-VEC formulation, thus demonstrating that the Het_B_ACT-VEC vaccine construct is antigenic and can stimulate memory CD4 T-cell recall responses in primary human cells. We further verified the ability of Het_B_ACT-VEC and VPs to stimulate primary and secondary immune responses using our MDDC–CD4 T-cell co-culture assay and PBMCs from healthy donors using intracellular cytokine staining flow cytometry (Fig. S5a, b). In this instance, Het_B_ACT-VEC was able to induce tumor necrosis factor (TNF)-α and IL-2 cytokine responses (average two-fold increase over media control) with only a low-level increase in IFN-γ. The VPs tested, especially VP 2, 4, and 5 were also capable of eliciting primary CD4 T-cell responses with the magnitude greater than that seen with Het_B_ACT-VEC. Again, no statistical difference in the generation of cytokine responses was observed between the different VPs and the Het_B_ACT-VEC formulation was detected (Fig. S5b).

In addition to VP5 and Het_B_ACT-VEC abilities to stimulate CD4 T cells, the formulations were evaluated for their abilities to stimulate exocytosis of Granzyme B (GzB), a potent proapoptotic granzyme associated with cytotoxic functioning. This was done using VP5 and Het_B_ACT-VEC pulsed PBMCs using ELISpot. Shown are the GzB cytotoxic response of two HIV^+^ volunteers upon stimulation with VP5 and Het_B_ACT-VEC (Fig. 5c). Volunteers 583 and 993 had a mean 25 and 35 GzB^+^ SFU/10^6^ PBMCs when stimulated with the media control (assay cutoff = 50 SFU/10^6^) while VP5 and Het_B_ACT-VEC had 161.7 and 127.5 GzB^+^ SFU/10^6^ and 113.3 and 105 GzB^+^ SFU/10^6^, respectively.

## Discussion

Over the past few centuries, empirically derived vaccines have slowly been replaced by rationally designed vaccines. During this time, killed/attenuated vaccines have demonstrated huge success in providing protection from a range of communicable diseases. However, safety concerns regarding insufficient attenuation and inactivation processes still remain. In the context of HIV-1 vaccines, attenuated versions of HIV raise serious ethical concerns due to the fear of reversion to virulence following vaccination or the potential for recombination with resident HIV following therapeutic vaccination.^26,27^ Methods to inactivate HIV-1, such as AT-2 treatment and UV irradiation, may result in a safe vaccine,^25^ but these conditions may also alter antigenicity and could affect the conformation of viral glycoproteins. As such, research into safer and stronger alternatives to the killed/attenuated HIV-1 has gained significant traction. This study describes the development of non-infectious VPs derived from infected patients and their reformulation into VLPs that are then used to produce a heterogeneous VLP formulation called Het_B_ACT-VEC. The Het_B_ACT-VEC is currently being tested as both a prophylactic and therapeutic vaccine. In the latter case, the Het_B_ACT-VEC is being used as a latency reversal agent as part of the “Shock and Kill Strategy”, the aim being to trigger proviral transcription and the elimination of infected cells.^15,28^

Our first prototype of this HIV-1 vector, termed VP, was derived from the pREC-nfl construct.^17^ HIV-1 mRNA is transcribed off pREC-nfl (near full length) from a minimal cytomegalovirus promoter, deleting the 5′LTR promoter/enhancer region such that transcription is initiated at the first nucleotide of the primer binding sequence. Aside from deleting the Tat-transactivating response element found within the 5′LTR, all other RNA regulatory sequences remain intact such that all HIV-1 mRNA species (un-spliced, singly spliced, and multiply spliced) are expressed, the full HIV-1 proteome translated, and VPs assembled and released from these cells.^17^ We have previously shown that the nfl VPs released from this system cannot initiate reverse transcription (i.e., no minus strong stop DNA synthesis), are non-infectious, and cannot be complemented by superinfection with a wild-type HIV-1.^17^

Despite rendering the lentiviral VPs non-infectious, the presence of nfl viral genomic RNA within vaccine formulations still poses a concern in vaccine development. Therefore, we introduced extensive mutations into the stem loop 1 (dS.1) of the RNA packaging element that strongly reduced genomic RNA encapsidation in VLPs, paralleling observations made by Clever and Parslow in 1998 with a laboratory-adapted HIV-1 clone.^21^ In our studies, the dS.1 mutations coupled with the deletion of the 5′LTR did not impact p24 content or RT activity in the VLPs but depleted gRNA encapsidation and resulted in non-infectious particles. Finally, during the amplification of the patient-derived HIV-1 genomes, we introduced a _262_RRK>AAH mutation into the active site of HIV-1 IN. In contrast to the nfl VPs, the dS.1/mutIN VLPs and Het_B_ACT-VEC (with Δ5′LTR/mutIN/dS.1) harbored a defective IN incapable of the dinucleotide cleavage, which ensured the nullification of viral infectivity.

In the absence of Gag-gRNA complexes, Gag can still oligomerize to produce VLPs of varying size and structure, whereas the inclusion of the full proteome (including the Env glycoprotein) appears to generate more uniform VPs, which was evident from our electron microscopic images on all formulations. While HIV VP formation is dependent only on Gag production, Env is required for virus entry into host cells and could elicit protective antibody responses. We propose that these fully processed wild-type VPs may be highly antigenic and could induce neutralizing responses, considering the viral proteins should be in a native “wild-type” structure, and can even undergo conformational changes related to viral entry, core deposition, and dissolution in the cell cytoplasm. However, it should be pointed out that not all VPs or VLPs will carry intact, native-like trimers nor will all the expressed Env become appropriately cleaved, and a fraction would certainly exist as monomers, dimers, and gp41 stumps.^29^ The development of a neutralizing antibody response would of course be highly beneficial for an anti-HIV prophylactic vaccine, enabling the neutralization of transmitted founder viruses at the earliest time points following virus exposure; however, anti-HIV antibody and neutralizing antibody responses would also be beneficial for therapeutic vaccines. In the latter case, it is envisaged that either an elicited neutralizing or antibody-dependent cell cytotoxicity response could aid therapeutic vaccination by blocking de novo infections occurring in response to latency reversal (Shock) and also in the eradication (Kill) of virally infected cells.^15,28^

While B-cell immunogens are important in providing protection through the generation of neutralizing and non-neutralizing antibody production, T-cell immune responses play an important role in controlling viral loads and priming B-cell responses. Therefore, it stands to reason that an efficacious prophylactic or therapeutic vaccine should elicit both T- and B-cell responses to either prevent infection at the level of the mucosa or eliminate infection through sterilizing immunity. Hence, it is important that our formulations can elicit both CD4 T helper cell and GzB cytotoxic functions in PBMC. Until such time that a broadly neutralizing antibody response can be successfully elicited through vaccination, anti-HIV cytotoxic responses could be highly beneficial in both prophylactic and therapeutic vaccine settings, i.e., in the elimination of infectious foci during the narrow “window of opportunity” and in controlling/eradicating productively infected cells within HIV-infected individuals, respectively.^15,30,31^ Attenuated viral vectors (e.g., ALVAC or Ad5) expressing more conserved HIV-1 proteins (e.g., Gag) or protein motifs (e.g., repeated strings of immunodominant/conserved HIV peptides) have been tested as CTL-based vaccines with moderate success in animal models but mostly failed in human clinical trials.^32^ Although many factors led to these vaccine failures, CTL escape mutations rapidly appear in the infecting HIV during both preventative and therapeutic vaccine trials. These studies, as well as the failure of most humoral based vaccines, have prompted us to construct and test a heterogenous HIV vaccine, which consist of “dead” HIV-1 particles lacking undesirably foreign vector components.

Our yeast recombination system produces vectors that harbor the same HIV-1 quasi-species diversity found within patient samples. By combining the subtype B HIV quasi-species from five patients, we have generated a heterogenous dS.1/mutIN VLP (termed Het_B_ACT-VEC). To further develop our therapeutic and cure-based vaccine strategies, we screen patients for subtype infection and then employ the respective ACT-VEC, currently being tested in ex vivo experiments with the aim of selecting the most effective vaccine modalities for use in a future clinical trial.

We are currently testing different heterogenous forms of this dS.1/mutIN VLP as (1) anti-HIV therapeutic/cure strategies, designed to elicit T-cell responses for activating the latent HIV pool and for reducing viral loads, and (2) a prophylactic vaccine designed to augment antiviral antibody titres and to harness CD4^+^ T-cell responses. The DNA vector pREC-nfl is also being tested as a DNA vaccine administered by intramuscular electroporation in animal models and may be very effective in combination with the VLP and Het_B_ACT-VEC for prime-boost therapeutic and prophylactic vaccinations. As described in a recent study, Het_B_ACT-VEC may also lead to latency reversal and elimination of much of the HIV-1 reservoir that remains in these HIV-infected patients receiving cART. In conclusion, our dS.1/mutIN VLP formulations appear safe in vitro, and we anticipate that VLPs and Het_B_ACT-VEC formulations will be safe for use in humans; however, this will need to be confirmed in future planned non-human primate studies and human clinical trials. The VLPs and Het_B_ACT-VEC also present the entire HIV proteome in the correct conformation, reflect the HIV-1 genetic diversity within infected individuals and, consequently, have wide range of uses.

## Materials and methods

### Ethics statement

For antigenicity studies, either HIV-positive volunteers were recruited from the HIV adult clinical St Mary's Hospital (Imperial College NHS trust), through a protocol approved by the NHS Health Research Authority (protocol number: 14SM1988) or healthy volunteer PBMCs were purchased from Canadian Blood Services under institutional REB approval (no: 106951). Written informed consent was provided by all HIV+study participants prior to the study start. PBMCs from HIV^+^ volunteers used in these studies had suppression of viremia to <50 copies HIV-1 RNA/ml for >6 months on ART. For VP, VLP, and Het_B_ACT-VEC production, HIV^+^ sera from five consenting HIV+ adult volunteers were obtained under internal review board approval (AIDS125) at Case Western Reserve University, (CWRU, USA). Methods were performed in accordance with relevant regulations and guidelines.

### VP and VLP vaccine production

All formulations were cloned using a similar protocol to that previously described and schematically depicted in Figure S1.^16–18^ Briefly, sera-derived viral RNA was isolated using a viral RNA Isolation Kit (Qiagen, USA) and reverse transcribed to cDNA (Agilent Technologies, USA) using two primers to generate a 5′ (5020R) and 3′ (1.R3.B3.R) fragment encompassing the entire HIV-1 genome. The two overlapping cDNA fragments were then PCR amplified in a nested PCR protocol using 5′ and 3′ primer pairs described in Table S1. The two fragments were then transfected into *S. cerevisiae* in a 1:1 ratio with 2 μg SacII linearized plasmid, pRECΔgag-U3/URA3. Yeast colonies were selected on complete medium lacking leucine (C−Leu) plates supplemented with fluoroorotic acid (FOA). The resulting plasmid vectors were isolated by an in-house yeast miniprep and used to transform bacteria to amplify the DNA plasmid for purification as described previously. It is important to note that the PCR products harbored the amplified patient quasi-species, and as such, >100 yeast colonies were removed from Leu−/FOA plates for bulk plasmid purification and eventual reconstitution of sample quasi-species. The resulting plasmid constructs were isolated and then used to transfect 293T cells (NIH AIDS Reagent Program) with Fugene 6 transfection reagent (Promega, USA) to produce viral VP and VLPs. This procedure of highly efficient yeast-based recombination/cloning followed by 293T transfections is believed to preserve the HIV-1 quasi-species population better than a similar approach using bacterial-restriction enzyme cloning. VP and VLPs were then purified by centrifugation through 100 KDa MWCO centrifuge tubes (Amicon, USA) and re-suspended in sterile phosphate-buffered saline (PBS).

### Vaccine quantitation and protein production assessment

VP and VLP production from 293T cells was monitored for transfection efficiency by p24 ELISA assay, provided under an MTA by the AIDS Vaccine Program, National Cancer Institute (NCI) at Frederick, MD, USA. A radioactive RT assay was also used to measure VP and VLP levels in cell-free supernatants as described previously.^18^ Viral proteins in formulations were also analyzed by western blot using NuPAGE Novex 3–8% Tris–Acetate Protein Gels (Thermo-Fischer Scientific) and a 1:100 dilution of heat-inactivated serum derived from SHIV-infected macaques, before addition of a 1:2000 dilution of goat anti-monkey IgG: horseradish peroxidase (HRP) (Bio-Rad). Samples were then developed with 3,3′-diaminobenzidine (DAB) SK-4100 (Vector Laboratories). For anti-p17 western blots, a 10–20% Novex Tris-Glycine Mini-Gel (Thermo Fisher, Ca) was used. Membranes were blocked and then stained for 2 h with 1:5000 dilution of polyclonal rabbit anti-p17 antibodies (NIH AIDS Reagent Program), The membrane was then incubated with goat anti-rabbit HRP (Abcam) at 1:2000 concentration and developed using DAB Liquid Substrate (Vector Laboratories).

### Size estimation of vaccine particles

VP and VLP size and particle distribution were measured using DLS with a Malvern Zeta-Sizer Nano (Malvern Instruments Ltd) at 25 °C. Briefly, purified VP and VLPs were diluted into 1 ml PBS and placed into 4.5 ml polystyrene analysis cuvettes (Fisher Scientific, CA). The intensity of laser light scattered by the sample preparations was measured at 173° to the incident beam. The data were analyzed using the proprietary Malvern software, DTS (Nano Version 5.0), supplied with the machine. The size distribution and the polydispersity were measured using non-invasive back scatter.

### NGS analysis of vaccine formulations

The C2–V3–C3 region of envelope was amplified by an external-nested PCR amplification using the primers forward EnvB and reverse ED14 (external) and forward E80 and reverse E125 (nested) using PCR cycle conditions as described previously.^33^ To prepare the amplicon library for 454 sequencing, fusion primers including the Roche 454 titanium key sequence and multiplex identifier (MID) sequence for forward and reverse primers followed by the template specific forward (E110) and reverse (E125) sequences were generated. The nested products were re-amplified with barcoded MIDs. The PCR products were run on a 1% agarose gel to verify the 406 bp size and then purified with the Agencourt AMPure XP bead system with a bead: DNA ratio of 0.7:1 according to the Roche manual. Following purification, PCR amplified sample libraries were quantified using the Quant-iT PicoGreen dsDNA Assay Kit (Invitrogen), diluted, and pooled together at 10^6^ molecules/µl for pyrosequencing as per Roche 454 instructions.

Following emulsion PCR (emPCR) at a ratio of 0.5 molecules of sample library per bead, 5 × 10^5^ enriched beads were loaded onto the titanium picotiter plate, which was run on the Roche 454 GS Junior instrument. Raw sequence data were extracted by the MID tag using a custom analysis pipeline. The 454 amplicon adapters were trimmed and sequences of <200 bp were discarded. Sequences were edited using BioEdit v7.2.5 and aligned using maximum likelihood methods (MUSCLE).^34,35^ Neighbor joining and maximum likelihood trees were constructed with SEAVIEW 4 and visualized with FigTree 1.4.2. Kimura genetic distance analysis within each sample were calculated using MEGA 6 and is expressed as substitutions per nucleotide (s/nt).^36,37^ Any genetic variants with hypermutations at homopolymeric tracts and/or appearing less than three times were removed from the analyses.

### Isolation of resting CD4^+^ T lymphocytes and MDDCs

CD4^+^ T lymphocytes were enriched from PBMCs by negative depletion (Miltenyi Biotec) using magnetic microbeads. To obtain immature DCs, PBMC were plastic adhered at 37 °C for 2 h. Adhering monocytes were washed to remove non-adherent cells and then differentiated into MDDCs by culturing in complete RPMI (10% fetal calf serum (FCS)+2 mM L-Glutamine) supplemented with GM-CSF and IL-4 (1000 and 500 U/ml, respectively) for 6 days.

### TZM-bl infectivity assay

The infectivity of VP and VLP preparations were estimated in TZM-bl cells (NIH AIDS Reagent Program) by luciferase quantitation of cell lysates (Promega, Madison, WI). Briefly, TZM-bl cells were seeded at 1 × 10^4^/well prior to addition of 50 ng/ml (based on p24) VPs and VLP formulations. After 48 h incubation, cells were washed with PBS and lysed with 100 μl of lysis reagent. A 50 μl volume was used for luciferase quantification in a Synergy H4 Hybrid microplate reader (BioTek Instruments, Inc., Burlington, VT) using 50 μl of luciferase reagent. The extent of luciferase expression was recorded in relative light units.

### Transmission electron microscopy

VP- and VLP-transfected 293T cells were collected in 15 ml tubes before pelleting at 1250 rpm for 10 min. Cells were washed with sodium cacodylate (pH 6.5) before re-suspending in 500 μl of 2.5% glutaraldehyde in sodium cacodylate. Supernatants were removed, and pelleted cells were re-suspended in 1% osmium tetroxide in sodium cacodylate for 1 h with shaking. Samples were then centrifuged and washed with deionized water. Dehydration was performed by re-suspending samples in 1 ml of increasing concentrations of acetone (30%, 50%, 70%, 90%, 95%, 100%) for 10 min each. Serial resuspensions with acetone: TEM Resin-Araldite EMbed 812 (2:1, 1:1, 1:2, whole TEM resin) followed, until all acetone was replaced by resin. Samples were then baked at 60 °C for 48 h and resin-embedded samples were cut into 70 nm wide sections using an UltraCut UltraMicrotome (Sorvall) before copper mesh mounting and staining with Uranyl Acetate. Samples were dried and stained with Lead Citrate before washing thoroughly with sterile water. Samples were air dried and then imaged on a Philips CM10 TEM.

### Antigenicity assays

Human IFN-γ and GzB enzyme-linked immunosorbent spot (ELISpot) assays (Mabtech, USA), as well as intracellular cytokine staining flow cytometry were carried out on MDDC–CD4^+^ T-cell co-cultures and PBMCs. ELISpot were carried out as per the manufacturer's instructions. Briefly anti-IFN-γ precoated plates or anti-GzB-coated plates were washed with sterile PBS and then blocked for 30 min using complete RPMI. Plates were again washed before addition of 1 × 10^6^ cells/ml CD4^+^ T cell or 106 lymphocytes from PBMCs. MDDC-stimulated CD4^+^ T cells and PBMCs were then incubated for 16 h to assess the number of HIV-specific CD4^+^ T cells and GzB-secreting cytotoxic cells. Unstimulated and 5 μg/ml phytohemagglutinin (PHA)/ionomycin (Iono) (Sigma, USA)-stimulated cells served as controls. To detect spots, biotinylated anti-IFN-γ or anti-GzB antibody was added at 1 μg/ml for 2 h before washing and incubating with streptavidin–HRP for 1 h. Plates were washed and 100 μl/well of TMB substrate was added. SFU were enumerated per 10^6^ cells using the ImmunoSpot S5 UV Analyzer (Cellular Technology Ltd., Cleveland, OH) and ImmunoSpot 5.0.9 software. Results are mean values (+/− SEM). For flow cytometric analysis of T-cell activation, CD4^+^ T cells were incubated for 2 h with ACT-VEC or VP-pulsed MDDCs before 4 h incubation with monensin. Samples were washed using FACs buffer (2.5% FCS in PBS) and surface stained with anti-CD3 (BD Bioscience, SK7) and anti-CD4 (Biolegend, RPA-T4) antibody. Samples were washed again before permeabilizing using a BD FACS Fix Perm Kit (Becton Dickinson, USA). The samples were then incubated with anti-IFN-γ (Biolegend, 45.B3), anti-TNF-α (eBioscience, Mab11), and anti-IL-2 (Biolegend, MQ1-17H12) antibody in FACS perm wash solution before washing in FACs buffer and then fixing in 1.5% methanol-free paraformaldehyde (Polysciences, USA) in PBS. Samples were analyzed on a FACS LSR II instrument with the FACS Diva software. Data analysis was performed with FlowJo (Treestar Inc., OR, USA).

### Statistics

Mann–Whitney non-parametric *U*-test was used to determine inter sample statistical significance where indicated. We considered *p* > 0.05 to be statistically significant. Based on previous studies, a group size of seven (*n* = 3) was required to be provide sufficient power in the ELISpot studies. As samples from all patients were handled in the same way, there was no randomization or blinding.

### Data availability

Supporting data for this study are available from the corresponding author upon reasonable request.

## Electronic supplementary material

Supplementary Information

## References

[1] Lederman MM (2016). A cure for HIV infection: “not in my lifetime” or “just around the corner”?. Pathog. Immun..

[2] Rerks-Ngarm S (2009). Vaccination with ALVAC and AIDSVAX to prevent HIV-1 infection in Thailand. N. Engl. J. Med..

[3] Cohen KW, Frahm N (2017). Current views on the potential for development of a HIV vaccine. Expert. Opin. Biol. Ther..

[4] Stephenson KE, D’Couto HT, Barouch DH (2016). New concepts in HIV-1 vaccine development. Curr. Opin. Immunol..

[5] Santra S (2012). Breadth of cellular and humoral immune responses elicited in rhesus monkeys by multi-valent mosaic and consensus immunogens. Virology.

[6] Wang S (2006). Polyvalent HIV-1 Env vaccine formulations delivered by the DNA priming plus protein boosting approach are effective in generating neutralizing antibodies against primary human immunodeficiency virus type 1 isolates from subtypes A, B, C, D and E. Virology.

[7] Pal R (2005). Polyvalent DNA prime and envelope protein boost HIV-1 vaccine elicits humoral and cellular responses and controls plasma viremia in rhesus macaques following rectal challenge with an R5 SHIV isolate. J. Med. Primatol..

[8] Lu S, Grimes Serrano JM, Wang S (2010). Polyvalent AIDS vaccines. Curr. HIV Res..

[9] Bowles EJ (2014). Comparison of neutralizing antibody responses elicited from highly diverse polyvalent heterotrimeric HIV-1 gp140 cocktail immunogens versus a monovalent counterpart in rhesus macaques. PLoS ONE.

[10] Kwong PD, Mascola JR, Nabel GJ (2013). Broadly neutralizing antibodies and the search for an HIV-1 vaccine: the end of the beginning. Nat. Rev. Immunol..

[11] Fischer W (2007). Polyvalent vaccines for optimal coverage of potential T-cell epitopes in global HIV-1 variants. Nat. Med..

[12] Gohain N (2016). Molecular basis for epitope recognition by non-neutralizing anti-gp41 antibody F240. Sci. Rep..

[13] Hansen SG (2013). Immune clearance of highly pathogenic SIV infection. Nature.

[14] Zhao, C., Ao, Z. & Yao, X. Current advances in virus-like particles as a vaccination approach against HIV infection. *Vaccines (Basel)* **4**, 2 (2016). 10.3390/vaccines4010002 PMC4810054 26805898

[15] Pankrac J, Klein K, Mann JFS (2017). Eradication of HIV-1 latent reservoirs through therapeutic vaccination. AIDS Res. Ther..

[16] Moore DM, Arts EJ, Gao Y, Marozsan AJ (2005). A yeast recombination-based cloning system to produce chimeric HIV-1 viruses and express HIV-1 genes. Methods Mol. Biol..

[17] Dudley DM (2009). A novel yeast-based recombination method to clone and propagate diverse HIV-1 isolates. Biotechniques.

[18] Marozsan AJ, Arts EJ (2003). Development of a yeast-based recombination cloning/system for the analysis of gene products from diverse human immunodeficiency virus type 1 isolates. J. Virol. Methods.

[19] Weber J (2013). Sensitive cell-based assay for determination of human immunodeficiency virus type 1 coreceptor tropism. J. Clin. Microbiol..

[20] Archer J (2012). Use of four next-generation sequencing platforms to determine HIV-1 coreceptor tropism. PLoS ONE.

[21] Clever JL, Parslow TG (1997). Mutant human immunodeficiency virus type 1 genomes with defects in RNA dimerization or encapsidation. J. Virol..

[22] Venner CM (2016). Infecting HIV-1 subtype predicts disease progression in women of Sub-Saharan Africa. EbioMedicine.

[23] Hammonds J, Wang JJ, Yi H, Spearman P (2010). Immunoelectron microscopic evidence for Tetherin/BST2 as the physical bridge between HIV-1 virions and the plasma membrane. PLoS Pathog..

[24] Iwabu Y (2009). HIV-1 accessory protein Vpu internalizes cell-surface BST-2/tetherin through transmembrane interactions leading to lysosomes. J. Biol. Chem..

[25] Choi E (2016). First phase I human clinical trial of a killed whole-HIV-1 vaccine: demonstration of its safety and enhancement of anti-HIV antibody responses. Retrovirology.

[26] Chakrabarti LA (2003). A truncated form of Nef selected during pathogenic reversion of simian immunodeficiency virus SIVmac239Deltanef increases viral replication. J. Virol..

[27] Learmont JC (1999). Immunologic and virologic status after 14 to 18 years of infection with an attenuated strain of HIV-1. A report from the Sydney Blood Bank Cohort. N. Engl. J. Med..

[28] Deeks SG (2012). HIV shock and kill. Nature.

[29] Arakelyan A (2017). Flow virometry analysis of envelope glycoprotein conformations on individual HIV virions. Sci. Rep..

[30] Lewis GK (2016). The first 24 h: targeting the window of opportunity for antibody-mediated protection against HIV-1 transmission. Curr. Opin. HIV AIDS.

[31] Deng K (2015). Broad CTL response is required to clear latent HIV-1 due to dominance of escape mutations. Nature.

[32] Schiffner T, Sattentau QJ, Dorrell L (2013). Development of prophylactic vaccines against HIV-1. Retrovirology.

[33] Nankya IL (2015). Defining the fitness of HIV-1 isolates with dual/mixed co-receptor usage. AIDS Res. Ther..

[34] Edgar RC (2004). MUSCLE: a multiple sequence alignment method with reduced time and space complexity. BMC Bioinformatics.

[35] Edgar RC (2004). MUSCLE: multiple sequence alignment with high accuracy and high throughput. Nucleic Acids Res..

[36] Gouy M, Guindon S, Gascuel O (2010). SeaView version 4: multiplatform graphical user interface for sequence alignment and phylogenetic tree building. Mol. Biol. Evol..

[37] Tamura K (2013). MEGA6: Molecular Evolutionary Genetics Analysis version 6.0. Mol. Biol. Evol..

