# Supplementary material for: A heterogeneous human immunodeficiency virus-like particle (VLP) formulation produced by a novel vector system
Source: NPJ Vaccines. 2018 Jan 19;3:2. doi: 10.1038/s41541-017-0040-6 (PMC5775397; doi:10.1038/s41541-017-0040-6)
Supplement: Supplementary file 1 — Supplementary Information [file 41541_2017_40_MOESM1_ESM.pdf]

Figure S1

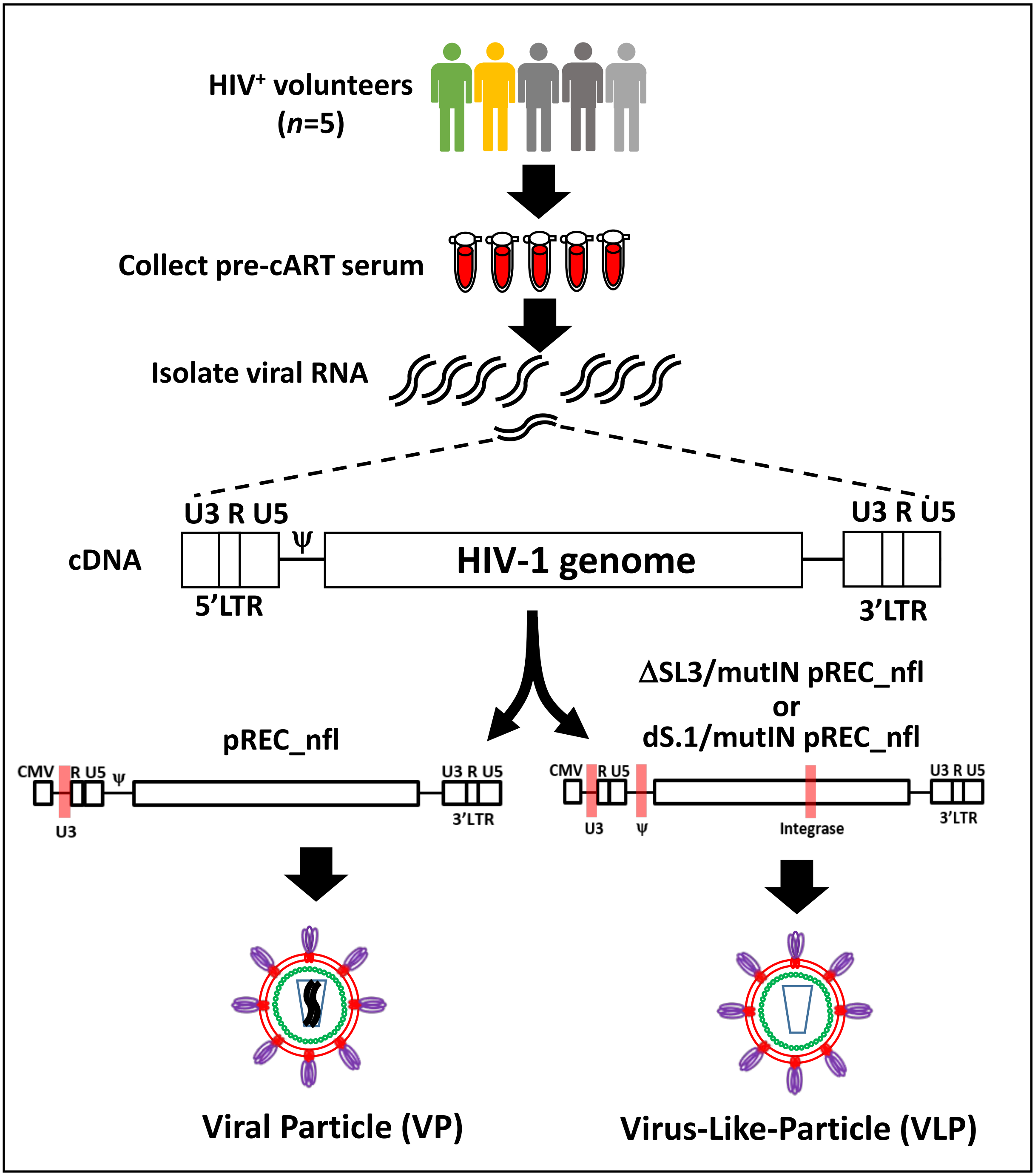

HIV+ volunteers
(n=5)
Collect pre-cART serum
Isolate viral RNA
cDNA
U3 R U5
Ψ
HIV-1 genome
U3 R U5
5'LTR
3'LTR
ΔSL3/mutIN pREC_nfl
or
dS.1/mutIN pREC_nfl
pREC_nfl
CMV
R U5
Ψ
U3 R U5
3'LTR
U3
CMV
R U5
U3 R U5
U3
Ψ
Integrase
3'LTR
Viral Particle (VP)
Virus-Like-Particle (VLP)

Figure S2

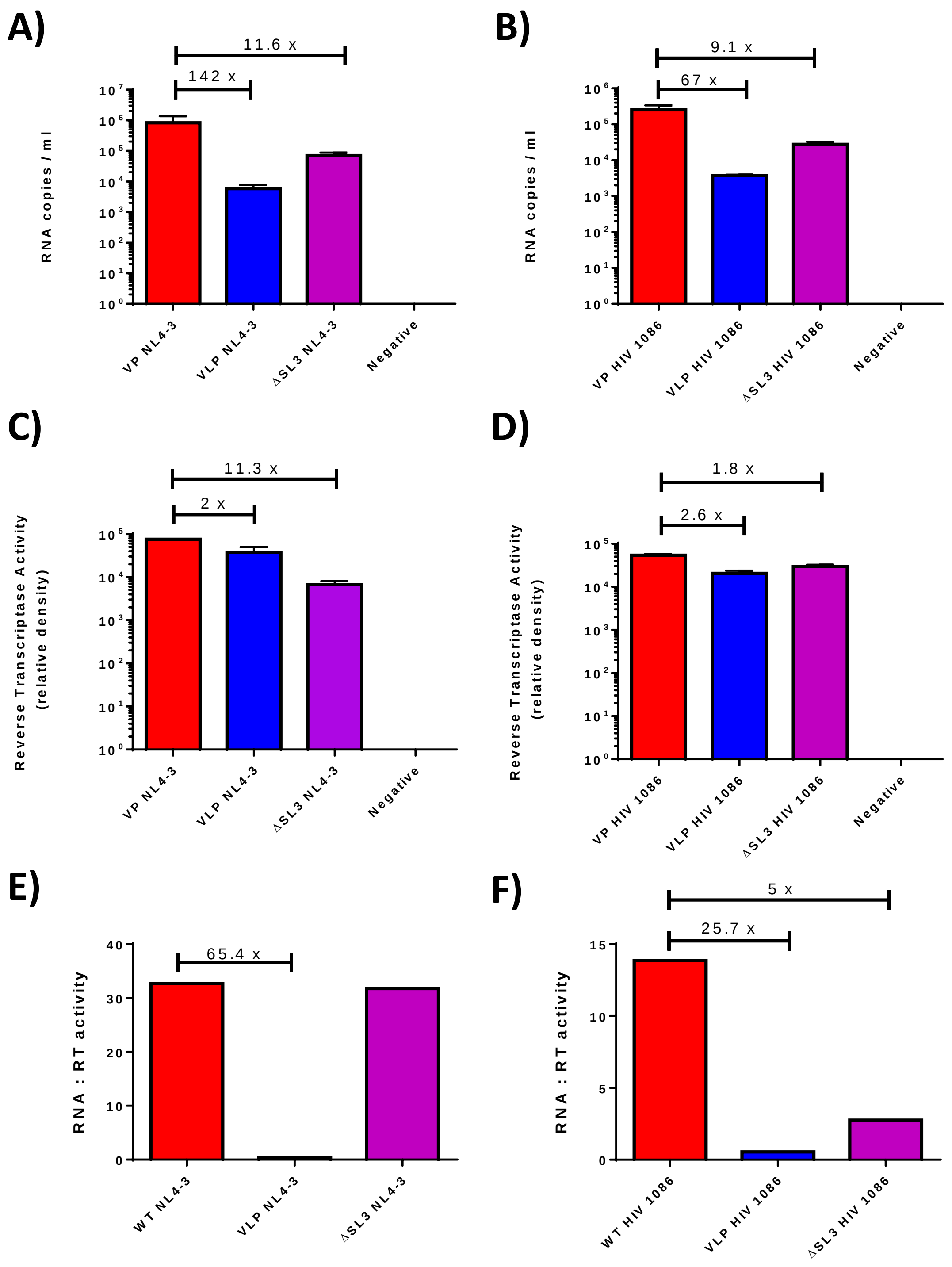
A)
11.6 x
142 x
RNA copies / ml
VP NL4-3
VLP NL4-3
ΔSL3 NL4-3
Negative
B)
9.1 x
67 x
RNA copies / ml
VP HIV 1086
VLP HIV 1086
ΔSL3 HIV 1086
Negative
C)
11.3 x
2 x
Reverse Transcriptase Activity
(relative density)
VP NL4-3
VLP NL4-3
ΔSL3 NL4-3
Negative
D)
1.8 x
2.6 x
Reverse Transcriptase Activity
(relative density)
VP HIV 1086
VLP HIV 1086
ΔSL3 HIV 1086
Negative
E)
65.4 x
RNA : RT activity
WT NL4-3
VLP NL4-3
ΔSL3 NL4-3
F)
5 x
25.7 x
RNA : RT activity
WT HIV 1086
VLP HIV 1086
ΔSL3 HIV 1086

Figure S3

A)

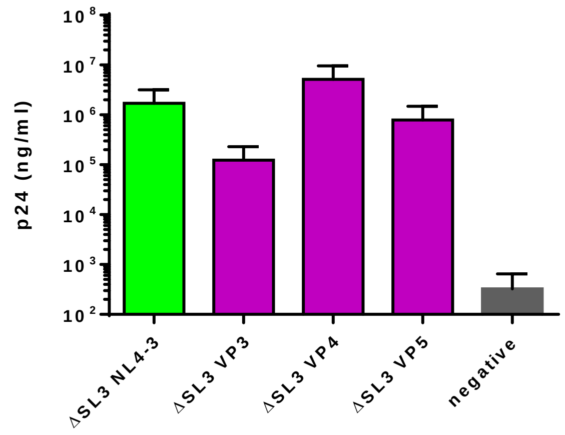

B)

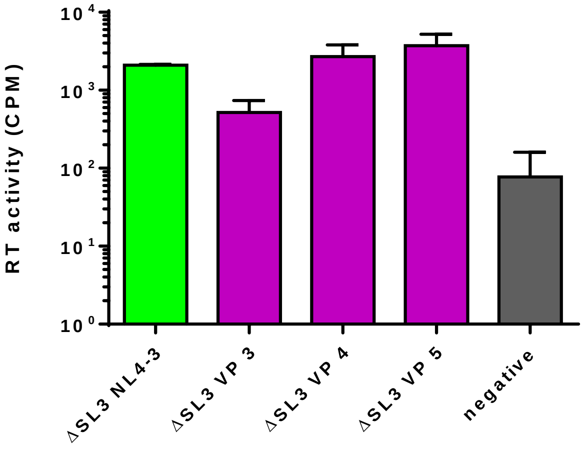

C)

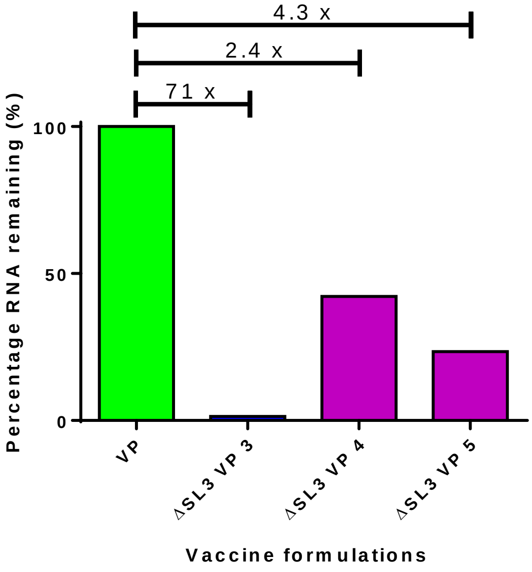

Figure S4

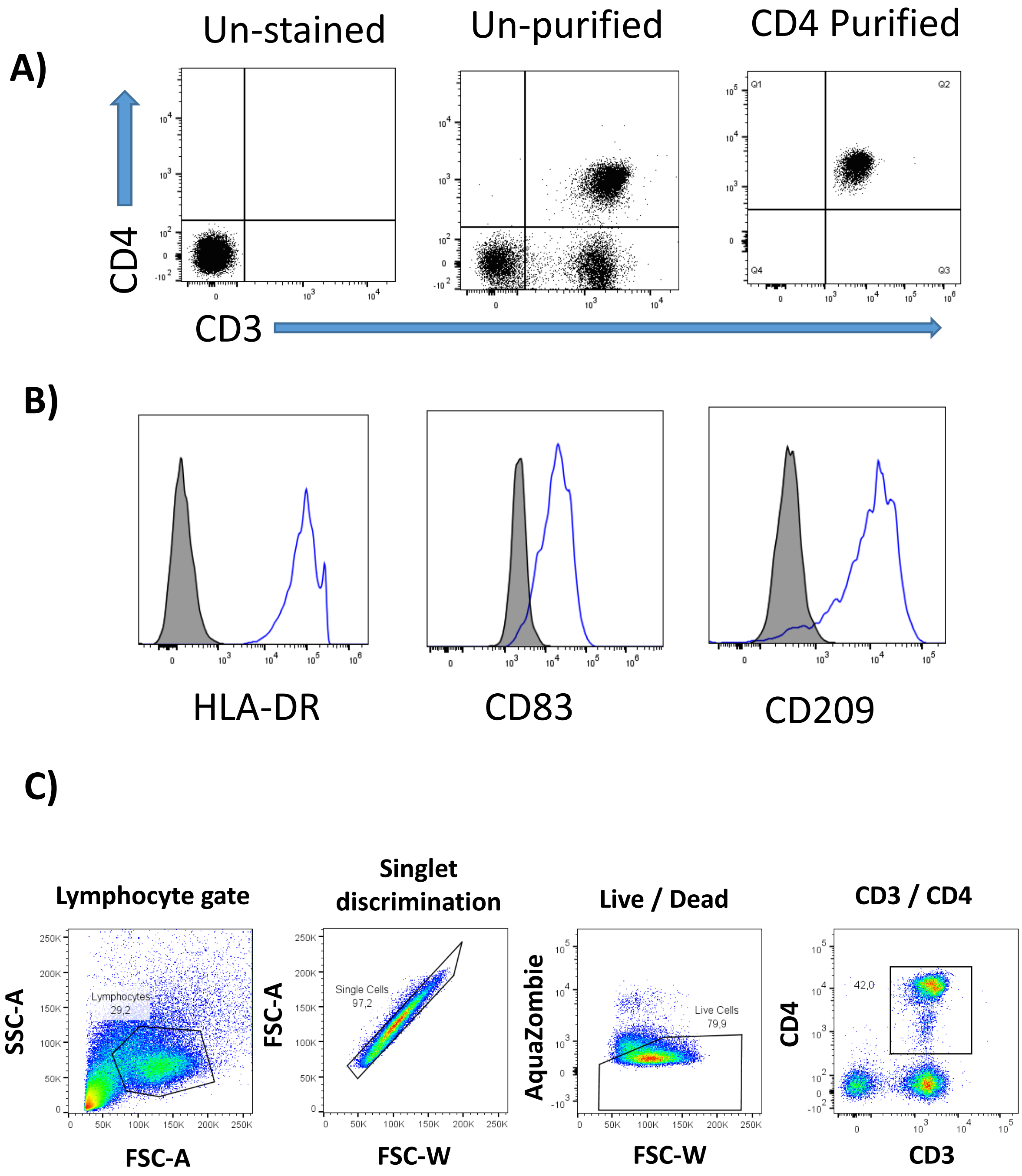

A)
Un-stained
Un-purified
CD4 Purified
CD4
CD3
Q1
Q2
Q4
Q3
B)
HLA-DR
CD83
CD209
C)
Lymphocyte gate
Singlet discrimination
Live / Dead
CD3 / CD4
SSC-A
FSC-A
Lymphocytes
29,2
FSC-A
FSC-W
Single Cells
97,2
AquaZombie
FSC-W
Live Cells
79,9
CD4
CD3
42,0

Figure S5

A)

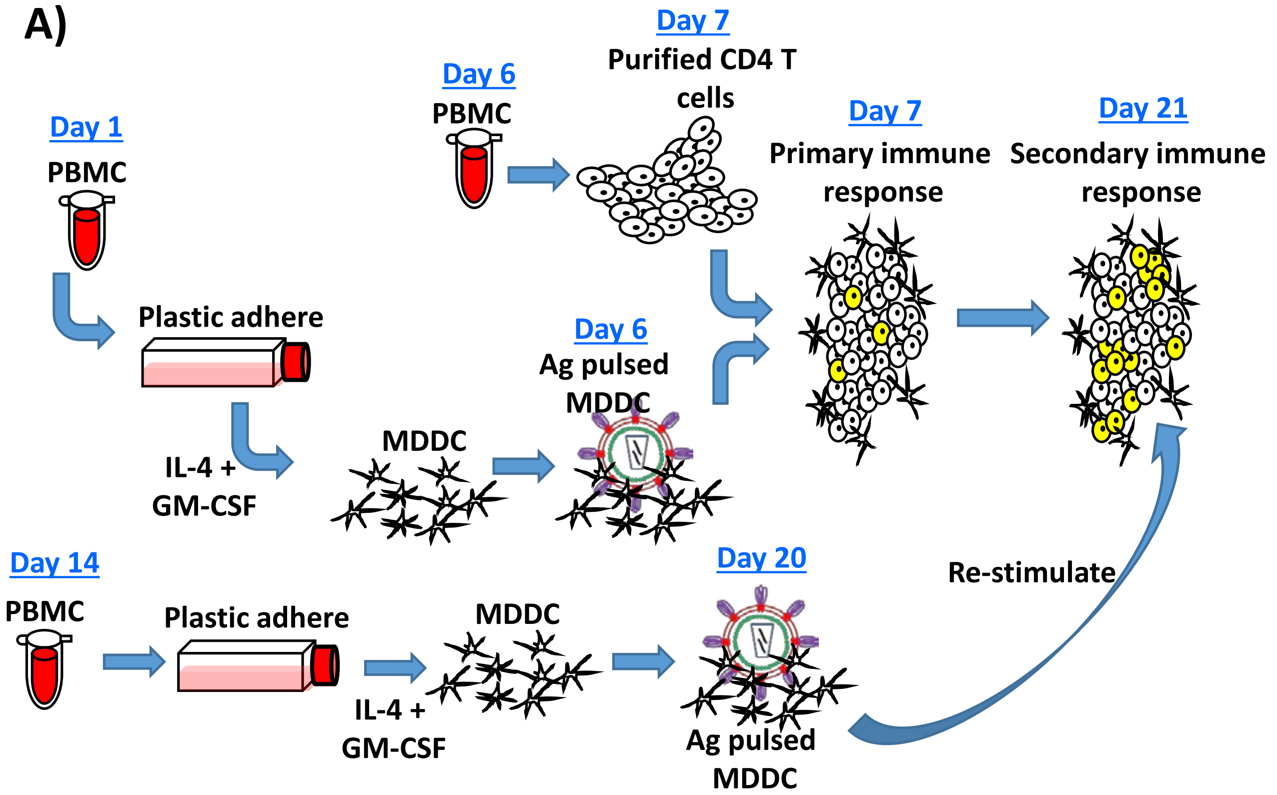

Day 1
PBMC
Plastic adhere
IL-4 +
GM-CSF
MDDC
Day 6
PBMC
Day 7
Purified CD4 T cells
Day 6
Ag pulsed MDDC
Day 7
Primary immune response
Day 21
Secondary immune response
Re-stimulate
Day 14
PBMC
Plastic adhere
IL-4 +
GM-CSF
MDDC
Day 20
Ag pulsed MDDC

B)

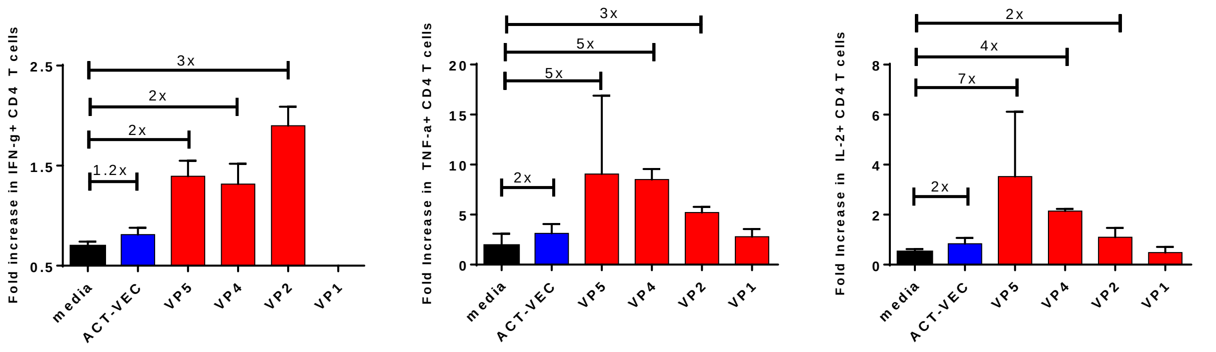

Fold increase in IFN-g+ CD4 T cells
0.5
1.5
2.5
3x
2x
2x
1.2x
media
ACT-VEC
VP5
VP4
VP2
VP1
Fold increase in TNF-a+ CD4 T cells
0
5
10
15
20
3x
5x
5x
2x
media
ACT-VEC
VP5
VP4
VP2
VP1
Fold increase in IL-2+ CD4 T cells
0
2
4
6
8
2x
4x
7x
2x
media
ACT-VEC
VP5
VP4
VP2
VP1

Table S1

	Primer	Sequence
**RNA - cDNA PCR**	1.R3.B3.R	ACTACTTGAAGCACTCAAGGCAAGCTTTATTG
	5202R	TTCTATGGAGACTCCCTGACCCAAATGCCA
**1st Round PCR**	517F	CTTAAGCCTCAATAAAGCTTGCCTTGA
	1.Int.R1	CTTGCCACACAATCATCACCTGCCAT
	1.Int.F1	ACAGCAGTACAAATGGCAGTATT
	9626R	CTTGAAGCACTCAAGGCAAGCTTTATTG
**2nd Round PCR**	Primer F	GGCGGCGACTGGTGAGTACGCCAAAAATTTTGACTAGCGGAGGCTAGAAGGAGAGAGATGG
	ΔSL3	GAGGCGAGGGGCGGCGACTGGTGAGTACGCCAGATGGGTGCGAGAGCGTCGGTATTAAGCG
	dS.1	GCAGGATTCGGCTTGCTGAAGCGCGCAGTCGTTCAGGCGAAGGGCGGCGACTGGTGAGTACG
	Int.R2	CAATCATCACCTGCCATCTGTTTTCCATAATCCCTGATGATCTTTGCGTGTGCTGCTGGCACTACTTTTATGTC
	Int.F2	TGGAAAGGTGAAGGGGCAGTAGTAATACAAGATAATAGTGACATAAAAGTAGTGCCAGCAGCACACGCAAAGATCATC
	47M	TCCCTAGTTAGCCAGAGAGCTCCC
**qRT-PCR**	5'LTR PA	CCCACTGCTTAAGCCTCAATAAAGC
	GagF	GGAGCTAGAACGATTCGCAG
	GagR	CTTACTTTTGTTTTGCTCTTC

Table S2

	Total number of sequence		Number of unique sequences		Average distance (s/nt)	
Volunteer	**VP**	**VLP**	**VP**	**VLP**	**VP**	**VLP**
**1**	2768	1641	74	31	0.022360	0.001608
**2**	1891	1240	50	22	0.005417	0.002445
**3**	4446	1874	98	39	0.002402	0.001327
**4**	1840	1521	49	23	0.001944	0.001701
**5**	3427	1498	54	38	0.001922	0.081980
**Average**	2874.4	1554.8	65	30.6	0.006809	0.017812

Supplemental information (SI):

Supplemental Figure Legends:

Supplemental Figure 1: Schematic of the VP and VLP cloning protocol: Schematic representation of the VP and VLP cloning strategy. Serum from consented HIV+ volunteers was used to extract viral RNA and generate two overlapping cDNA fragments. The cDNA is then used in a nested PCR reaction to generate two overlapping fragment of viral DNA for recombination in yeast using our in-house developed pREC_Δgag-U3 recombination vector. In the nested PCR, mutagenic primers dS.1 or ΔSL3 insert extensive nucleotide substitutions in stem loop 1 of the RNA packaging sequence or delete stem loop 3. Successful recombination in yeast and subsequent bacterial amplification results in pREC_nfl or ΔSL3/mutIN pREC_nfl or dS.1/mutIN pREC_nfl DNA for use in VP and VLP production. Areas where modifications to the viral genome were introduced are indicated by red shading. Graphics depicted in this figure were generated by the authors.

Supplemental Figure 2: Comparison of the impact on RNA packaging by differently engineered RNA packaging mutations. pREC_nfl plasmid constructs using NL4-3 and HIV 1086 as the vector backbone were constructed with mutations in stem loop 1 (SL1, VLP) or deletion of stem loop 3 (ΔSL3). The ability of the SL1 or SL3 modifications to impact RNA packaging in NL4-3 and HIV 1086 were assessed using 293T cell transient transfections and qRT-PCR with a gag primer set (**A and B**). The mean RNA copes/ml (+/-SEM) are shown. Viral particles (VP) lacking modifications to the RNA packaging sequence and media were used as positive and negative control respectively. Reverse transcriptase activity in the NL4-3 and HIV 1086 VP and VLP formulations were assessed using radioactive reverse transcriptase assay and densitometry readings on radiographical film (**C** and **D**). The ratio of RNA to reverse transcriptase activity was calculated for the SL1 VLP and ΔSL3 modified NL4-3 and HIV 1086 formulations (**E** and **F**).

Supplemental Figure 3: The deletion of stem loop 3 (ΔSL3) causes RNA packaging defects in viral particles. The ΔSL3 mutagenic primer was used in the nested PCR reaction for the five cDNA's derived from HIV+ volunteer samples. Of the five ΔSL3 VPs, only ΔSL3 VP 3, 4 and 5 resulted in successful recombinants in yeast. The DNAs were then used to transfect bacteria and subsequently transfect 293T cells. The purified ΔSL3 VP 3-5 were assayed for p24 content (**A**) in culture supernatants by ELISA and for reverse transcriptase activity using a radioactive RT assay (**B**). The percentage RNA in ΔSL3 VP 3-5 were determined, by first isolating viral RNA and generating cDNA, before using qRT-PCR and a gag primer set to quantify viral genomic levels (**C**). Results for ΔSL3 VP 3-5 RNA levels are shown as percentage RNAs relative to the volunteer matched and unmodified VPs.

Supplemental Figure 4: Cryopreserved PBMC from fully consented HIV+ volunteers under continuous anti-retroviral treatment where used in these studies. PBMC were defrosted and $CD4^+$ T cells isolated by magnetic isolation and negative selection. The purify of the isolated $CD4^+$ T cells was determined by live/dead cell gating prior to $CD3^+$ and $CD4^+$ gating. A representative flow cytometry plot is shown (**A**). PBMC were defrosted to generate MDDC. Initially, PBMC were allowed to plastic adhere in tissue culture flasks monocytes before incubation for 6 days in the presence of IL-4 and GM-CSF. Immature DC were then phenotypically characterized by flow cytometry using HLA-DR, CD83 and CD209 antibodies (**B**). To evaluate Het_B_ACT-VEC and VP immunogenicity, the formulations were used to pulse the prepared MDDC overnight, followed by co-incubating the MDDC with autologous $CD4^+$ T cells. The flow cytometry gating strategy used to characterize define the $CD4^+$ T cells used in the immunogenicity studies is shown (**C**).

Supplemental Figure 5: Cryopreserved PBMC from healthy donor volunteers where used in these studies. A schematic of the assay used to determine primary and secondary immune reactivity towards ACT-VEC and VPs 1,2,4 and 5 is shown (**A**). The ability of Het_B_ACT-VEC and VP1,2,4 and 5 to induce primary and secondary immune

responses were assessed by flow cytometry for IFN-γ, IL-2 and TNF-α using an LSRII and both FACS Diva and Flowjo software (**B**).

Supplemental Table 1: Primers used in VP and VLP production.

Supplemental Table 2: Characterization of pREC_nfl sequence diversity. The five pREC_nfl DNA's derived from HIV+ volunteers (VP) were assessed by next generation sequencing (NGS). The corresponding four dS.1/mutIN VLPs (1-4) and Het_B_ACT-VEC (VLP 5) DNA's were also characterized after nested PCR induced RNA packaging site disruption (dS.1) and nucleotide substitutions in Integrase (mutIN). The total number of sequences derived from NGS runs using the VP and VLP plasmid DNA's, the number of unique sequences within each DNA sample used to generate VP and VLP samples and the calculated average distance (substitutions/nucleotides) in each DNA preparation is shown. To determine the number of unique sequences, the total number of sequences in the samples were collapsed using on-line Galaxy software. The genetic distance was calculated using MEGA 6 software.
